# Use of thermal analysis coupled with differential scanning calorimetry, quadrupole mass spectrometry and infrared spectroscopy (TG-DSC-QMS-FTIR) to monitor chemical properties and thermal stability of fulvic and humic acids

**DOI:** 10.1371/journal.pone.0189653

**Published:** 2017-12-14

**Authors:** Patrycja Boguta, Zofia Sokołowska, Kamil Skic

**Affiliations:** Department of Physical Chemistry of Porous Materials, Institute of Agrophysics, Polish Academy of Sciences, Lublin, Poland; VIT University, INDIA

## Abstract

Thermogravimetry–coupled with differential scanning calorimetry, quadrupole mass spectrometry, and Fourier-transform infrared spectroscopy (TG-DSC-QMS-FTIR)–was applied to monitor the thermal stability (in an N_2_ pyrolytic atmosphere) and chemical properties of natural polymers, fulvic (FA) and humic acids (HA), isolated from chemically different soils. Three temperature ranges, R1, 40–220°C; R2, 220–430°C; and R3, 430–650°C, were distinguished from the DSC data, related to the main thermal processes of different structures (including transformations without weight loss). Weight loss (ΔM) estimated from TG curves at the above temperature intervals revealed distinct differences within the samples in the content of physically adsorbed water (at R1), volatile and labile functional groups (at R2) as well as recalcitrant and refractory structures (at R3). QMS and FTIR modules enabled the chemical identification (by masses and by functional groups, respectively) of gaseous species evolved during thermal decomposition at R1, R2 and R3. Variability in shape, area and temperature of TG, DSC, QMS and FTIR peaks revealed differences in thermal stability and chemical structure of the samples between the FAs and HAs fractions of different origin. The statistical analysis showed that the parameters calculated from QMS (areas of m/z = 16, 17, 18, 44), DSC (Max_DSC_) and TG (ΔM) at R1, R2 and R3 correlated with selected chemical properties of the samples, such as N, O and COOH content as well as E2/E6 and E2/E4 indexes. This indicated a high potential for the coupled method to monitor the chemical changes of humic substances. A new humification parameter, H_TD_, based on simple calculations of weight loss at specific temperature intervals proved to be a good alternative to indexes obtained from other methods. The above findings showed that the TG-DSC-QMS-FTIR coupled technique can represent a useful tool for the comprehensive assessment of FAs and HAs properties related to their various origin.

## Introduction

Monitoring changes in humic substances (HS) in soils is of key importance for agriculture [1,2]. HS perform a fundamental role in the environment as they influence the chemical, physical and biological properties of soil: air-water relations, porosity, viscosity, temperature, sorption and buffer capacity, and activity of microorganisms. HS have an influence on plant growth, as they serve as a source of micronutrients, nitrogen, phosphorus, and carbon. However, HS exhibit an undefined structure with a high variability of functional groups, aromaticity, molecular weights, solubility, and reactivity in the environment. Thus, HS concentration should be studied in soil and water in parallel to their chemical and physicochemical properties. This could be problematic since the high degree of their heterogeneity can make the use of conventional analytical methods for HS ineffective; new analytical tools, however, promise the delivery of comprehensive results related to the qualitative and quantitative assessment of the chemical, physicochemical, and spectral properties of HS.

Thermogravimetric analysis (TG) has been used in the study of organic compounds in soils, biochars, sediments or sludges—mainly to assess the hydrologic parameters or organic matter content and loss, e.g., during wildfires [3–6]. In the evaluation of humic compounds, TG has previously been used mainly to assess structure stability [7–10]. Studies on HS have revealed the presence of a few weight loss steps during the temperature increase, presumably attributable to labile, recalcitrant, and refractory structures [11,12]. However, these TG results have failed to provide a precise and full qualitative and quantitative identification of the kinds of thermal processes and the type of structures released during HS decomposition, humification, and reactivity.

The development of TG, coupled with other instrumental techniques, initially with the differential thermal analysis (DTA), differential scanning calorimetry (DSC), and, currently, with the evolved gas analysis (EGA), has marked a breakthrough in the thermal analysis of organic macromolecules. DTA has been commonly used in the analysis of organic substance [13–15] although this technique is characterized by low accuracy, fails to record polymorphic transformations or the synthesis of new phases or some other processes involving structure destruction. It only allows for the approximate evaluation of the direction of thermal changes in the samples [16].

TG-DSC coupling is much more precise compared to DTA. It allows the quantitative measurement of heat in individual thermal transformations and, thus, permits a description of all thermal processes–Including those without the mass change [2,17]. The main techniques used for EGA include gas chromatography (GC), mass spectrometry (MS) and Fourier-transform infrared spectroscopy (FTIR) [16,18]. The data obtained through these methods can be used to identify the gaseous species emitted by the sample, by studying their mass or their vibrational spectra over a wide range of temperatures [19]. For instance, the TG-MS combination offers a qualitative and quantitative description of gases evolved through thermal decomposition as well as exposing their relation to the weight loss of the sample. The coupling of TG and FTIR spectroscopy provides a very useful tool for determining functional groups and observing changes in aliphaticity and aromaticity during the decomposition process [20]. MS or QMS (quadrupole mass spectrometry) reveals a higher sensitivity and specificity for chemical compounds; however, FTIR can be used for handling heavy molecules and, consequently, is useful in the studies of pyrolysis [19].

There have been some studies carried out using the coupled systems TG-MS, TG-FTIR, TG-DSC/DTA in HS analyses [14,21–23]. However, these double-coupled systems still cause some inconveniences. The TG-DSC results can be difficult to interpret due to a high complexity of the organic matter. Fusions, decomposition and polymerizations can occur together with a temperature increase, resulting in the superposition of endothermic and exothermic effects [12]. MS cannot resolve compounds with the same m/z values while FTIR detects only those substances that change dipoles.

The combination of all the techniques described above could enable a more comprehensive analysis of HS samples. Until now, no studies have been conducted on soil HS (here fulvic acids (FAs) and humic acids (HAs)), using a real-time, simultaneous analysis in a TG-DSC-QMS-FTIR system under the conditions of pyrolysis at different thermal steps, both in the FTIR and QMS analysis. A slow thermal degradation of organic matter in N_2_ atmosphere is still poorly understood in contrast to oxidation [12]. There are very few reports about the application of TG-DSC-MS-FTIR coupled techniques to other materials such as coals [24], polymers [18], or biomass [25]. Moreover, only a limited number of investigations have dealt with the relationships between the parameters measured in the thermogravimetric-coupled techniques and the parameters calculated from other methods, whereas thermal stability could be related to the surface properties of biomaterials and could serve as a good indicator of HS quality.

Therefore, the aim of this work was to use the coupled TG-DSC-QMS-FTIR method under N_2_ for the analysis of HAs and FAs of different soils in order to: a) determine the stability of chemical structures, b) describe the physicochemical properties of HS, such as functional groups, aromaticity, and aliphaticity with the differentiation into FAs and HAs fractions, c) determine the degree of humification, d) assess the lability/recalcitrance of humus carbon. The study discussed in this paper attempts, for the first time, to perform a statistical analysis between parameters calculated from TG-DSC-QMS-FTIR and those determined with other methods that typify humus compounds. Moreover, TG-DSC-QMS-FTIR parameters were determined for different temperature ranges, and a description of the individual thermal process/reaction (according to the first DSC derivatives) is provided.

## Materials and methods

### Fulvic and humic acids

FAs and HAs were isolated from chemically different soils (A-horizons) by the procedure recommended by the International Humic Substances Society (IHSS) [26]. Selected physicochemical and chemical properties of the soils and HAs were described previously [27]. FAs were analyzed in terms of the same chemical parameters as HAs: the elemental composition (C, H, N) was determined using a CHN 2400 analyzer (Perkin Elmer). The oxygen content was calculated from the difference: O% = 100%—(C%+N%+H%) and then the atomic ratio of C/N was estimated. The carboxylic (COOH) and phenolic groups (OH) were measured via the method proposed by Dragunowa and Kucharenko [28]. The E2/E6 and E2/E4 indexes were determined as ratio of absorbance measured respectively at 280 and 665 nm as well as at 280 and 465 nm of FAs (40 mg dm^-3^) in 0.05 M NaHCO_3_ using a UV-Vis spectrometer (Jasco V-520) [29]. The Kumada parameter (ΔlogK) was calculated as the difference between decimal logarithm of absorbance at 400 and 600 nm: ΔlogK = logA400—logA600. However, it should be emphasized that original description and classification of the above parameter is related only to humic acids. The values for fulvic acids in this paper are only indicative. Three replicates were performed for each treatment and the results were averaged.

### Coupled thermal analysis: TG-DSC combined with evolved gas analyses QMS and FTIR

Thermal decomposition of FA and HA samples with evolved gas analyses (EGA) were carried out using the coupled TG-DSC-QMS-FTIR system. All modules of the above system recorded signals simultaneously in real time during the temperature increase.

### TG-DSC module

TG and DSC characteristics were obtained on a STA 449 F3 Jupiter thermogravimeter (Netzsch, Germany) equipped with a thermobalance and TG–DSC sample carrier. A total of 15 mg of the sample was placed in an Al_2_O_3_ crucible and then the whole system was degassed and filled with N_2_ (99.999% purity) three times. Afterwards, the sample was progressively heated from 40 to 800°C at a heating rate of 10°C min^−1^ under N_2_ atmosphere of 70 ml/min (carrier gas: 50 ml/min and balance protective gas: 20 ml/min). The gaseous compounds released from the sample were continuously transported through the isothermal transfer lines at 250°C to prevent condensation of the compounds, to the QMS and FTIR modules and analyzed in real time of entire measurement. The thermobalance, on which the analyzed sample was placed, continuously recorded mass change during decomposition of the sample (i.e., from gas release) at each point of the temperature increase from 40 to 800°C. TG characteristics were obtained as a percentage weight loss related to the initial mass as a function of temperature. The first derivative of the TG (dTG) was calculated to highlight the temperature of the most intensive mass loss.

DSC results were recorded as changes in heat flow in the sample during temperature increase. Temperature ranges of thermal effects, R1 = 40–220°C; R2 = 220–430°C; and R3 = 430–650°C, were established for all samples, based on the first derivatives of DSC. The dDSC derivative was used instead of the dTG because of much more detail. Weight loss (ΔM) was estimated and averaged for each temperature range and for total temperature range from TG characteristics, as described in the previous paragraph for TG measurement.

The humification index, H_TD_ (the subscript standing for ‘thermal decomposition’), based on ΔM values at the R1, R2 and R3 temperature ranges, is proposed by authors herein and calculated as:

$$H_{TD}=\frac{\Delta M_{R1}+\Delta M_{R2}}{\Delta M_{R3}}$$ (1)

Maximal values of energetic effect within R1, R2 and R3 ranges (Max_DSC_) were read from DSC curves at temperatures corresponding to characteristic points on dDSC curves. The total exoenergetic effect (Hexo) was expressed as the area under the DSC curve in the R2+R3 temperature region (220–650°C). TG-DSC module was calibrated before analyses for temperature and sensitivity, using standard metals with known melting points. Data collection together with signal correction for baseline drift was carried out with Proteus software.

### Modules of evolved gas analyses (EGA): QMS and FTIR

During sample decomposition in TG-DSC module, the quadrupole mass spectrometer QMS 403C Aëolos (Netzsch, Germany) was used to continuously detect ion currents (IC) originating from particular gaseous products as m/z signals in the range of 10–300 amu. The acquisition of MS data was achieved with Aeolos 7.0 software. MS final characteristics were obtained as IC of given m/z as a function of temperature. In order to compare relative intensity of m/z peaks for various HAs and FAs (semiquantitative analysis), the m/z lines were normalized to the maximum of total IC [19,30]. Afterwards, integrated peak areas of the main m/z lines were determined for the R1, R2 and R3 temperature intervals and compared within studied samples providing information about the amount of released gases. It needs to be pointed out that only the peak areas of the same m/z for different HAs and FAs can be compared. In case of various m/z signals only shape and characteristic temperatures can be compared.

As noted above, the second part of gaseous products was continuously transported from TG-DSC to TG-FTIR module, which was equipped with liquid-nitrogen cooled MCT detector and coupled with FTIR main module (Tensor 27, Bruker). Spectra were recorded in 3D mode (absorbance/temperature/wavenumber) at the range of 600–4000 cm^-1^ with 4 cm^-1^ resolution. Acquisition of data was controlled by OPUS software. For each HA and FA sample, 2D FTIR spectra were “extracted” from 3D spectra at three temperatures corresponding to temperatures of maximal DSC effect within the R1, R2 and R3 temperature intervals.

### Statistical analysis

Statistical analysis of relationships between the chemical properties of FAs, HAs and the selected parameters obtained from TG-DSC-QMS-FTIR system was performed using Student's t-test. The significance of the correlation coefficients was analyzed at the p = 0.05. The assumption of normal distribution for all data used in statistical calculations was verified by the Shapiro-Wilk test.

## Results and discussion

### Fulvic and humic acids

The studied FAs and HAs exhibited a wide diversity in terms of their chemical properties. Detailed characteristics of the samples as well as their origin are presented in Table 1.

**Table 1:** Chemical properties of soil HAs and FAs (data for HAs previously reported in Boguta and Sokołowska [27]).

HA/FA origin			HA/FA no	C	H	N	O (S)	C/N	COOH	OH	E2/E6	ΔlogK	E2/E4
Soil type	Soil location	Soil pH H_2_O/KCl		(atomic %)					(cmol kg^-1^)				
Stagnic Luvisol (Grey-brown soil)	50°38N /22°41’E	5.94/5.28	FA1	37.3	34.0	1.34	27.3	27.9	632	229	147.7	1.07	14.26
			HA1	43.8	35.2	2.13	18.8	20.5	260	239	41.8	0.75	6.77
Haplic Fluvisol (Alluvial soil)	51°09’N /22°59’E	6.56/5.89	FA2	36.9	33.0	1.59	28.5	23.1	690	179	200.2	1.13	19.14
			HA2	40.9	35.6	3.27	20.2	12.5	321	329	28.8	0.70	5.37
Mollic Gleysol (Black Earth)	50°22’N /23°39’E	7.88/7.89	FA3	39.2	33.2	2.22	25.3	17.7	561	224	211.2	1.16	15.21
			HA3	39.4	36.7	2.72	21.2	14.5	424	246	67.5	0.89	9.54
Haplic Cambisol (Brown Soil)	51°23’N /22°35’E	4.45/3.57	FA4	38.7	33.6	1.08	26.6	35.9	649	290	283.2	1.20	17.54
			HA4	36.6	39.4	2.27	21.7	16.2	411	330	31.6	0.67	5.55

The studied FAs were characterized by typical values of elemental composition for this fraction of HS. FA3, originating from black earth, exhibited the highest content of N and C as well as the lowest O content and C/N atomic ratio, which brought this sample closer to the values for the HA fraction and provided evidence of a higher degree of humification. In contrast, a considerably high C/N ratio was revealed by FA4, which according to Tan [31] indicated the lowest progression in humification processes of this sample. The above conclusions were coherent with parameters calculated on the basis of UV-Vis spectra. The highest values of E2/E6 were determined for FA4, which confirmed its low humification degree, i.e. a high amount of lignin-like compounds in relation to amount of compounds being in the final stage of humification. The lowest values of E2/E6 and E2/E4 were exhibited by FA1, which was probably connected with a high amount of medium and strongly transformed structures, abundant and highly condensed aromatic compounds as well as higher molecular weight of this sample. FAs showed the number of COOH groups about 2–3 times higher than OH groups. The highest content of COOH and OH structures was determined for FA2 and FA5, respectively, which suggested high sorption capabilities of these samples.

In case of HAs, part of the results are depicted and discussed in more detail in Boguta and Sokołowska [27]. Briefly, the elemental composition of HAs indicated significant diversity, especially in C content. The highest amount of this element and simultaneously the lowest O, H and N content were measured for HA1. The C/N ratio was the highest for HA1, which suggested the lowest humification stage for this sample within HA fraction. Both H and especially N content in HAs were higher than in the FAs samples (opposite to O concentration).

One of the possible explanation of ~2 times higher N content for HAs in comparison to FAs can be polymer biodegradation theory, assuming that HAs can be degraded to FAs with simultaneous loss of N [31]. The C/N values of HAs when compared to FAs were much lower, which mainly resulted from the differences in N content. In the group of the studied HAs the highest E2/E6, E2/E4 values were in HA3, suggesting significant participation of lignin-like structures in relation to structures in terminal and medium stage of humification. The ΔlogK values indicated the studied HAs were medium and weakly humified, but more humified than corresponding FAs. The number of OH groups was comparable for FAs and HAs. However, the COOH content was much lower for HAs indicating weaker complexation abilities.

### TG and DSC analysis

**Fig 1:**
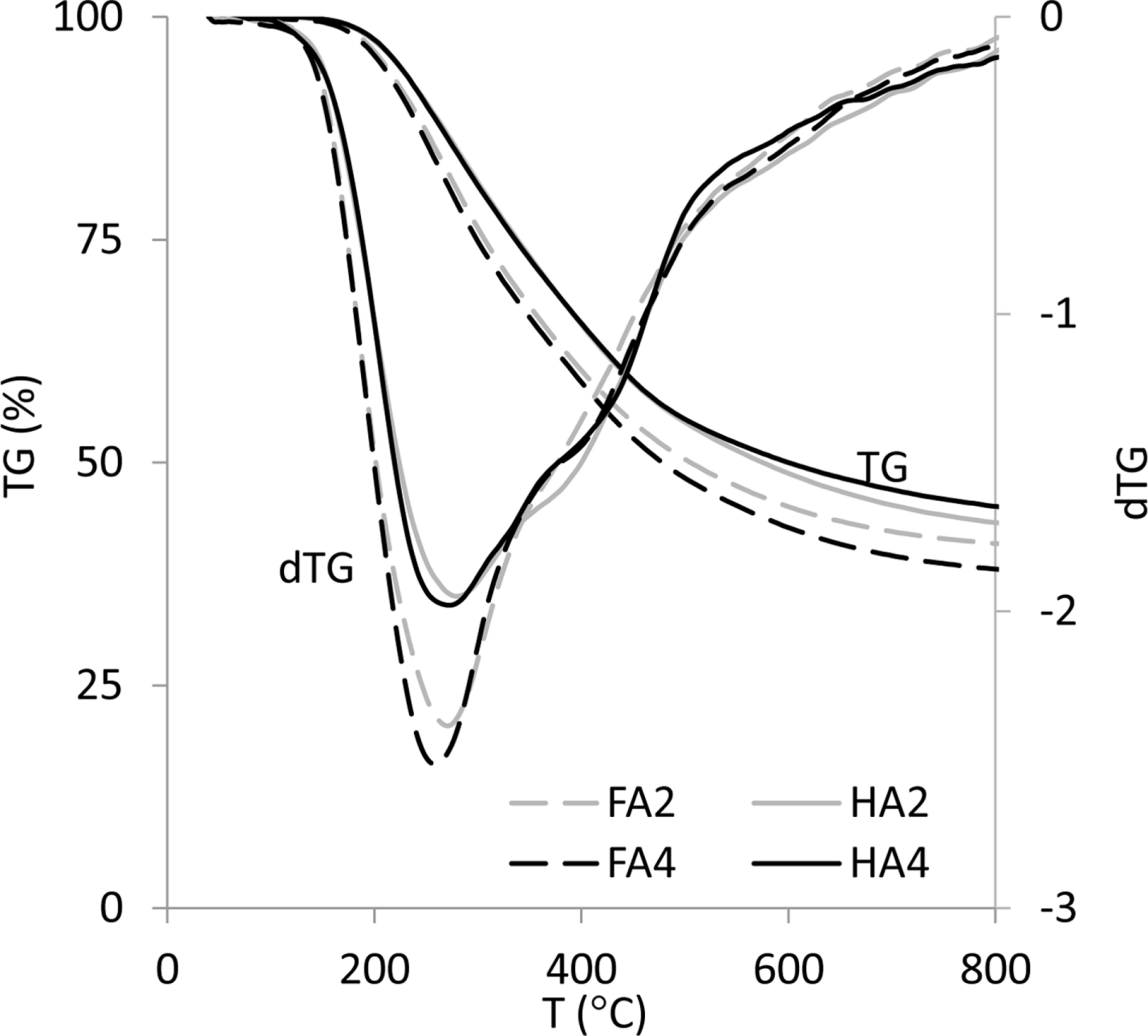
Thermogravimetric curves (TG) with first derivatives (dTG) of exemplary FAs and HAs.

Thermograms of FAs and HAs fractions (TG characteristics) as well as first derivatives (dTG) are shown in Fig 1 as well as in supporting information: S1 Fig.

TG curves of all the studied samples showed gradual weight loss in function of temperature increase. In total analyzed temperature range (R_tot_) the weight loss was higher for FAs (56.2–59.2%) than for HAs (40.3–53.3%), suggesting a higher number of thermolabile groups in the structure of FAs as compared to HAs. The highest weight loss at R_tot_ was exhibited by FA4, which could be connected with the lowest humification degree (the highest values of C/N), whereas the lowest weight loss was showed by HA1, a sample characterized by significant humification degree, the highest C content and the lowest COOH amount. Incomplete decomposition of the samples was consequence of the pyrolytic character of degradation. Detailed values of R_tot_ for all HAs and FAs are displayed in Table 2.

**Table 2:** TG, MS and DSC characteristics of soil HAs and FAs at temperature ranges R1, R2 and R3.

	Thermogravimetry					Mass Spectrometry												Diff. Scann. Calorimetry			
Sample	ΔM					A m/z = 16			A m/z = 17			A m/z = 18			A m/z = 44			Max_DSC_			Hexo (DSC)
	(%)					(A·s)												(mW/mg/min)			(J/g)
	R1	R2	R3	Rtot	H_TD_	R1	R2	R3	R1	R2	R3	R1	R2	R3	R1	R2	R3	R1	R2	R3	(R2+R3)
**FA1**	6.8	36.8	13.3	56.8	18.7	81	638	432	229	839	589	853	2388	1403	218	1613	269	0.41	1.80	3.55	1190
**HA1**	4.5	26.7	9.2	40.3	13.6	22	526	456	239	973	612	922	2780	1405	89	940	52	0.29	1.59	2.71	1122
**FA2**	7.6	35.3	13.3	56.2	17.7	103	677	338	303	835	553	1017	2386	1369	278	1735	340	0.34	1.66	3.52	1095
**HA2**	4.8	32.3	13.3	50.4	15.3	15	540	535	181	914	674	772	2565	1489	93	1068	171	0.47	1.38	3.24	1226
**FA3**	6.8	36.7	13.2	56.6	18.5	69	626	501	244	892	625	895	2581	1430	215	1583	266	0.48	1.46	4.39	969
**HA3**	4.8	34.0	14.6	53.3	15.2	30	555	562	186	899	682	745	2562	1597	114	1135	161	0.34	1.07	2.76	1015
**FA4**	7.3	37.7	14.2	59.2	19.3	77	679	513	270	837	591	901	2400	1423	240	1768	317	0.40	1.82	3.80	1425
**HA4**	4.7	33.8	13.3	51.8	15.8	20	536	527	162	890	623	783	2537	1515	98	1051	133	-	1.13	2.38	1268

ΔM: mass change; A: area of m/z; Max_DSC:_ maximum of energetic effect in given temperature range read out from dDSC; Hexo (DSC)–heat of the exoenergetic effect in 220–650°C; R1: temperature range 40–220°C; R2: temperature range 220–430°C; R3: temperature range 430–650°C; R_tot_: temperature range 40–650°C; H_TD_: humification index based on thermal decomposition.

The intensity of weight loss was different depending on temperature, due to decomposition of different chemical bonds. These changes were revealed in the dTG characteristics as two degradation rate peaks/shoulders (Fig 1). A similar peak pattern was seen in other studies [11,15,24,32]. The strongest changes were observed on dTG above 150°C with a maximum at the range of 250–270°C, indicating decomposition of simple and labile structures [16]. The intensity of this process was higher for FAs than HAs, which was the evidence of the higher number of structures with lower bond energy in FAs. The dTG curves at higher temperatures also revealed other changes corresponding to decomposition of consecutive structures, but these peaks were not well resolved.

**Fig 2:**
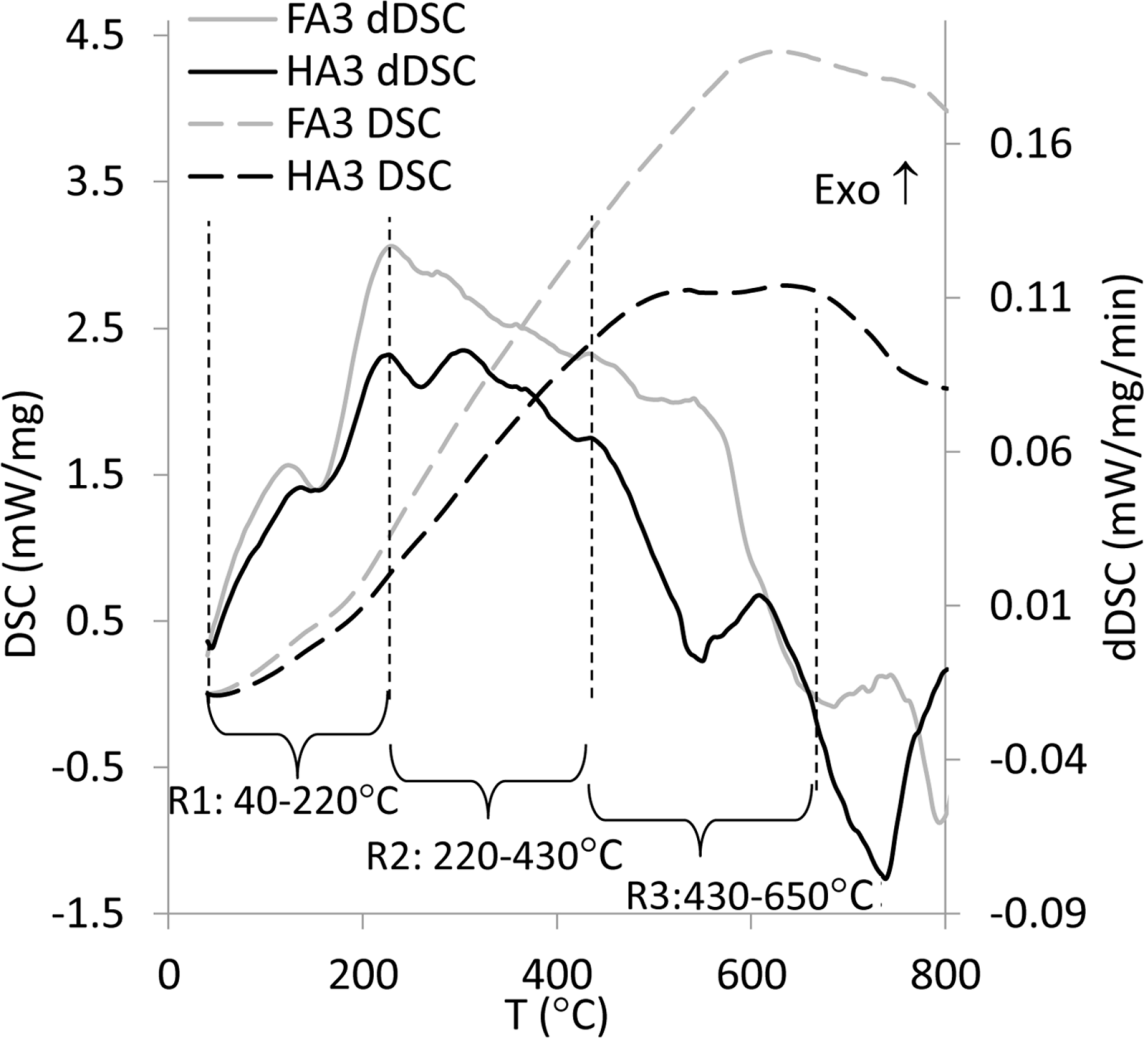
Curves of differential scanning calorimetry (DSC) and first derivatives (dDSC) of exemplary FA and HA.

DSC characteristics and especially their first derivatives (dDSC) provided more detailed information, due to higher sensitivity and the fact that they display all reactions with heat transfer—even those without weight loss (as opposed to TG and dTG). DSC and dDSC characteristics are presented in Fig 2 as well as in supporting information: S2 and S3 Figs.

All dDSC curves contained a peak or shoulder system consisting of: (a) an endothermic effect with maximum temperature of ~150°C and (b) two distinctive exothermic regions, the first one at temperature of ~270°C and a second one at ~550°C. This common peak pattern for the studied samples could result from similar origin (soil) and affiliation of the samples to the group of humic substances; however, according to Esteves and Duarte [8] and Zhang et al. [10], the number of characteristic peaks can be varied for different organic matter depending on origin. The peaks found on dDSC might have represented different nature organic carbon structures and functional groups in HS [2,32]. Taking into account the width of three found peaks, three corresponding temperature intervals were defined for all studied samples: R1 at 40–220°C, R2 at 220–430°C and R3 at 430–650°C. The region above 650°C was not considered in these studies due to possible slight influence of traces of inorganic compounds [33]. Both weight loss calculated from TG curves (ΔM) and maximal values of thermal processes heat from DSC curves (Max_DSC_) were found in temperature intervals R1, R2 and R3 (see Table 2). Additionally, experimental data of ΔM for thermal effects at R1, R2 and R3 were collated in Table 3 with data from the literature for humic and fulvic acids of different origin, analyzed at various conditions.

**Table 3:** Experimental and reference data of weight loss at individual temperature ranges for humic and fulvic acids of different origin and for various conditions of thermogravimetric measurements.

Sample	Conditions	Step1	ΔM1	Step2	ΔM2	Step3	ΔM3
		(°C)	(%)	(°C)	(%)	(°C)	(%)
**Experimental data of the present study**							
Soil HAs	N_2,_ TG-DSC-MS-FTIR	40–220	4.5–4.8	220–430	26.7–34	430–650	9.2–14.6
Soil FAs	N_2_ TG-DSC-MS-FTIR	40–220	6.8–7.6	220–430	35.3–37.7	430–650	13.2
**Reference data**							
Aquatic HAs ^[^^11^^]^	N_2_, TG	40–100	4.6–7.7	270–440	53.6–59.1	n.d.	n.d.
Aquatic FAs ^[^^11^^]^	N_2_, TG	40–100	5.4–10.1	270–440	44.7–63.2	n.d.	n.d.
Gley soil HS ^[^^34^^]^	N_2_, TG-DTA	40–190	17.1	190–435	18.63	435–567	6.44
Stream sediment HAs ^[^^35^^]^	N_2_, TG	60–90	~6.7	260–350	16–30	350–900	
Tropical Soils HS ^[^^22^^]^	He, TG-DSC-MS	<180–220	n.d.	180–325	5.2–9.1	325–540	11.1–28.1
Temperate Soils HS ^[^^22^^]^	He, TG-DSC-MS	<160–180	n.d.	160–540	5.1–11.9	180–540	8.9–28.1
Chromic Luvisol FA ^[^^32^^]^	O_2_, TG	30–105	2,25–8,75	105–350	35.2–75.3	350–600	24.7–64.8
Chromic Luvisol HA ^[^^32^^]^	O_2_, TG	30–105	5,67–15,81	105–350	28.2–64.8	350–600	35.2–71.8
Lignite HAs ^[^^2^^]^	Air, TG-DSC	<250	6.4–7.9	250–440	70–80.5	445–510	14.2
Mangrove swamp sediments FAs ^[^^10^^]^	Air, TG	30–107	10.8–13.0	101–418	33.9–40.5	371–600	46.5–55.3
Gley soil HS ^[^^34^^]^	Air, TG-DTA	40–173	12.5	173–627	39.45	627–954	31.9

The endothermic effect (within R1) was mainly attributed to the evaporation of water physically adsorbed and structurally incorporated into organic particles (e.g., crystal and interlayer water molecules) [2,11,23,32]. The effect was similar in all samples, showing a small weight loss (4.5–7.6%). Slight temperature shifts of these peaks’ maxima indicated differentiation in water molecules binding with other structures [14]. A small share of decomposition processes of organic carbon was also possible in R1 range, thus it was worth to note that exothermic processes could slightly overlap endothermic effects.

Distinctive changes were found in R2 and R3 temperature ranges. The ΔM within R2 interval was the highest and could have been associated with decomposition of simple and labile organic structures like functional groups (carboxylic, methylene, alcoholic, aldehydes, amides, amines and phenol groups), polysaccharide C–O bonds and simple aromatics (biodegradable components) [2,7,10,32]. In this region, dehydration of aliphatic structures took place as well [23]. A similar decomposition step was also reported during a pyrolysis experiment on humic substances by Giovanela et al. [11]. Weight loss of FAs in R2 interval was greater than in case of HAs (Table 2). This indicated FAs’ higher number of polar and oxygen-containing functional groups compared to HAs [10].

The R3 region was attributed to decomposition of N-compounds, long chain hydrocarbons, more refractory, aromatic, polyaromatic and polyheterocyclic structures as well as cleavage of C–C bonds [10,13,32,36,37]. Weight loss of HAs at R3 interval reached values similar to FAs’ weight loss. This fact might seem to be surprising, due to the higher amount of nitrogen and aromatic structures in HAs, which should result in elevation of the weight loss at R3 in relation to FAs. However, Giovanela et al. [11] described the possibility of aromaticity increasing upon heating, especially for HAs. Such a process could cause a shift of decomposition temperature to the much higher values and thereby it could explain the relatively low weight loss of HAs at the R3 interval. Zhang et al. [10] stated there were a higher number of long aliphatic chains in HAs than FAs, and also the possibility of their condensation to cyclic structures and aromatization upon heating. In our studies, subtle disruptions without significant weight loss on dTG and pronounced changes in dDSC characteristics above 500°C (Figs 1 and 2) may suggest the presence of such processes. In this case DSC analysis proved to be more useful than dTG characteristics (showing only weight changes) or DTA analysis (much less accuracy).

Values of the H_TD_ index were lower for all studied HAs in relation to the group of FAs (see Table 2). Taking into account that humic acids display in general a higher humification degree than the fulvic acid fraction, it can be assumed that low values of the H_TD_ index can correspond with strong humification while high values with weak humification. This statement has been confirmed in the present study by statistical analysis (high positive correlation of H_TD_ index with E2/E6, E2/E4 and ΔlogK, as described in a later section). The highest H_TD_ was shown by FA4, a sample that simultaneously exhibited the highest weight loss in the R2 region. It can be concluded that significant number of labile structures in FA4 determined its low humification, i.e., low aromaticity and molecular mass. In general, the H_TD_ index revealed significant differentiation within the studied samples, which can make it useful for assessment of HS structure. Taking into account that H_TD_ index changed for HAs from 13.6 to 15.8 and for FAs from 17.7 to 19.3, we can conclude that HS samples can be described as: strongly humified (typically for humic acids) when H_TD_ are 13.6–15.8, weakly humified when H_TD_ are 17.7–19.3 and moderately humified when H_TD_ values are ~16. The lowest and the highest values of above ranges can additionally correspond with the strongest and the weakest humification degree within the specific group of HS.

Values of Max_DSC_ at R3 were higher than at R2 and at the same time higher for FAs than for corresponding HAs (Table 2). It was probably due to the presence of aromatic groups, which are more thermally recalcitrant and need higher temperatures (R3) for cleavage [36]. Total exothermic effect (Hexo) was the highest for FA4 –sample with the highest ΔM at R2 (Table 2), which suggested that the number of labile functional groups determined also the total heat of the thermal degradation.

### Evolved gas analysis: TG-DSC-QMS

**Fig 3:**
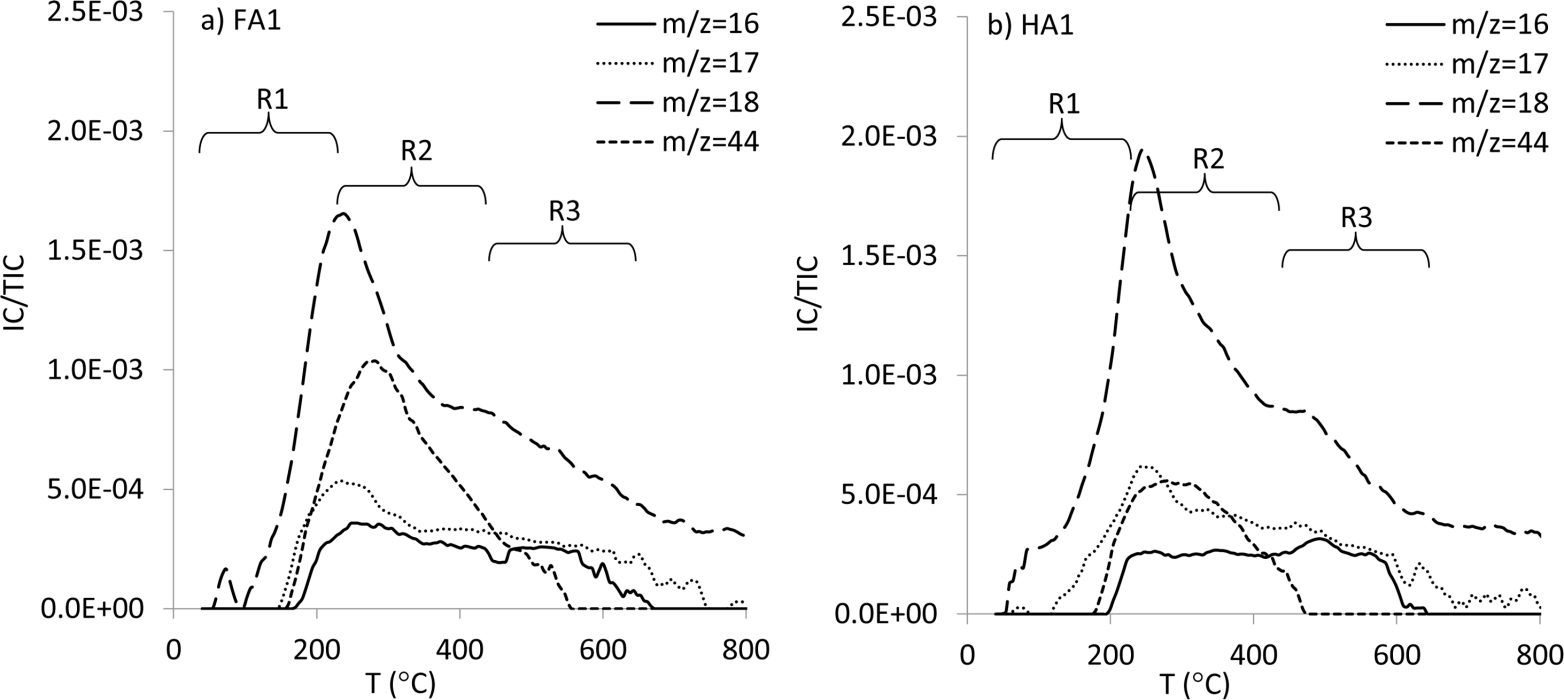
The distribution of the ion current (IC) of the main m/z intensities for (a) FA1 and (b) HA1.

The mass spectra of the gaseous products of HAs and FAs thermal decomposition showed clearly pronounced signals of m/z = 16, 17, 18 and 44. The distribution of the above ion currents (IC) for exemplary FA and HA is illustrated in Fig 3. Moreover, others m/z signals were measured as well: m/z = 12, 13, 15, 20, 26, 27, 30. However, these IC values were not considered, due to their insignificant levels. In this paper, areas of main m/z were calculated for R1, R2 and R3 temperature intervals (Table 2). IC derived from m/z = 16 could correspond to CO_2_, -NH_2_, CH_4_, CO and to a lesser extent to traces of chinones and O in N-, and S-oxides. The m/z = 17 can be attributed to the presence of N-compounds and H_2_O molecules; however, the main signal from H_2_O molecules was observed as m/z = 18. The intensities recorded as m/z = 44 were related to CO_2_ release [18,38].

In case of all studied FAs and HAs, the R1 temperature region was dominated by H_2_O release (m/z = 18) mainly from moisture [19,39], however carbon compounds were also detectable in this region, already from ~150°C. Surprisingly, the maximum of m/z = 18 took place at the beginning of R2 temperature region and this signal showed right-sided extension to the higher temperatures. The shape of m/z = 18 line at the beginning of R2 temperatures was similar to m/z = 17, suggesting release of the same kind of compound, and which could be attributed, at such high temperatures, to interlayer water molecules, as well as to water from dehydration reactions and decomposition of crystal water [9,40]. Arenillas et al. [19] reported that H_2_O evolution above 300°C could be also produced by the decomposition of various oxygen-containing groups, mainly OH groups. In our studies, m/z = 18 maximum for FAs was observed at lower temperatures (233–247°C) than for HAs (246–249°C), which indicated that water molecules binds by FAs were weaker than by HAs. However, the area of m/z = 18 at R2 was higher for HAs compared to FAs, showing the higher amount of water released. It should be noted that shape of m/z = 17 and m/z = 18 lines began to vary slightly at the highest temperatures of the R2 region, showing weak, relative elevation of m/z = 17. This indicated that besides H_2_O, N-compounds could also be released above 400°C.

The maximum of m/z = 44 (CO_2_) was also in the R2 region (276–296°C), but it was slightly shifted to the higher temperatures and was also much wider in relation to maximum of m/z = 18. This fact indicated distinct processes responsible for release of both gases and especially on high differentiation of chemical structures incorporating carbon. CO_2_ evolution in R2 region was mainly derived from aliphatic and aromatic carboxyl and carboxylate groups [19]. An interesting observation was that area of m/z = 44 at R2 region was always higher for FAs than HAs despite higher C content in the structure of HAs (see Table 1). The lowest m/z = 44 area was measured for HA1, which was the sample with the highest C content. These data show that considerable part of HAs carbon was refractory for temperatures from the range of 220–430°C.

QMS results at R3 temperature interval reflected decomposition of more refractory compounds like polyheterocyclic, polyaromatic structures and long chain hydrocarbons. The IC values of particular m/z lines were, in general, lower than in R2, however interesting changes in their courses were observed. First of all, an m/z = 18 slight signal (H_2_O) was still present and a weak maximum or shoulder was visible at about 440°C, especially for HAs, which suggested release of H_2_O molecules from the most thermally stable functional groups. CO_2_ (m/z = 44) at R3 could derive from stable ether-structures, quinones and oxygen-bearing heterocycles [19]. Interestingly, CO_2_ presence was detected by QMS to the higher temperatures for FAs (~580°C) than for HAs (~520°C). The shape of m/z = 16 was at the R3 region elevated in relation to m/z = 44 and was detected to the higher temperatures (even up to 700°C) in comparison to signals of m/z = 44. The above results demonstrated that m/z = 16 at R3 reflected also release of other compounds than CO_2_, possibly the products of thermal decomposition of long chain hydrocarbons, heterocyclic and aromatic compounds, like -NH_2_ and CH_4_. De la Rosa et al. [21] and Lopez-Capel et al. [30] in studies of fire-affected organic matter suggested that a relative increase in stable forms of C and N above 500°C might be associated with polycondensation processes. In our investigations nitrogen compounds in R3 region were also confirmed by slight elevation of m/z = 17 in relation to the shape of m/z = 18. These compounds could derive from amine, pyrrole, indole and carbazole groups [19]. The above N and C compounds were also probably the reason for higher areas of m/z = 16 at R3 for HAs than for FAs (the opposite as in R1 and R2, where the area of m/z = 16 was always higher for FAs). The lowest area of both m/z = 16 and m/z = 17 at R3 was revealed by FA2, which indicated the lowest number of recalcitrant structures.

### Evolved gas analysis: TG-DSC-FTIR

**Fig 4:**
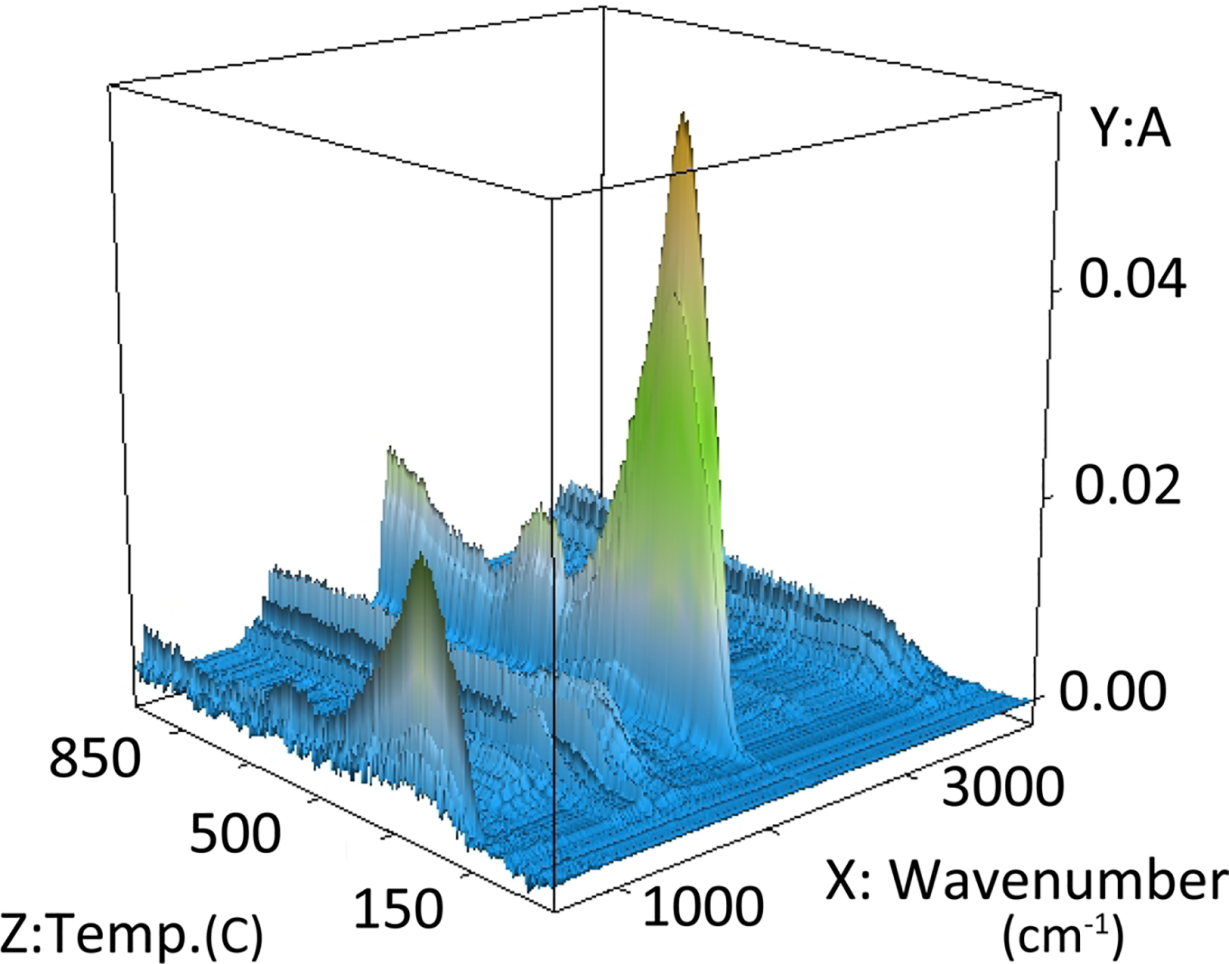
FTIR 3D spectrum of the evolved gases of FA3 as a function of temperature.

The 3D FTIR spectra revealed significant changes in intensities of particular absorption bands of decomposition products of HS as a function of temperature. Exemplary 3D spectrum of the evolved gases is shown in Fig 4. Main absorption bands are described in Table 4.

**Table 4:** Frequencies and relationships between the main, characteristic FTIR bands of the gaseous products of thermal decomposition at the maximum temperatures of the DSC effects in R1, R2 and R3 temperature ranges.

Assignment	Wavenumber (cm^-1^)	Description of bands							
O-H	3500–4000	Water, phenols and alcohols: H-bonded -OH, intermolecular bonded OH: stretching vibrations							
N-H	3500–3300	Nitrogen compounds: asymmetric stretching vibrations							
C-H	3016–3020	Aliphatic structures: -CH_2_, -CH_3_, CH_4_: asymmetric stretching vibrations							
CO_2_	2400–2240	Carbon dioxide							
C = O	1900–1650	Carbonyl compounds (ketones, aldehydes, acids): asymmetric stretching vibrations							
C = C, C = O	1650–1530	Aromatic compounds, carbonyl compounds: asymmetric stretching vibrations							
C = C, C-H	1550–1400	Aromatic compounds: symmetric stretching vibrations, aliphatic structures: bending vibrations							
C-H, C = O	1390–1250	Aliphatic structures, carbonyl compounds: symmetric stretching vibrations							
O-H	<1200	Tertiary, secondary, primary alcohols							
		**Relationship between absorbance intensities in R1, R2 and R3 temperature ranges**^**a**^							
		**FA1**	**HA1**	**FA2**	**HA2**	**FA3**	**HA3**	**FA4**	**HA4**
OH	~3780	R3> R1> R2	R3> R1 = R2	R3> R1> R2	R1> R3> R2	R1> R3 = R2	R3>R2	R3> R1 = R2	R3> R1> R2
OH	~3590	R2>R3>R1	R2>R3>R1	R2>R3>R1	R2>R3 = R1	R2>R3 = R1	R2>R3	R2>R3>R1	R2>R3>R1
CH_4_	~3016	R3	R3	R3	R3	R3	R3	R3	R3
CO_2_	~2360	R2>R3>R1	R2>R3>R1	R2>R3>R1	R2>R3>R1	R2>R3>R1	R2>R3	R2>R3>R1	R2>R3>R1
COOH	~1790	R2>R3>R1	R2>R3>R1	R2>R3>R1	R2>R3 = R1	R2>R3 = R1	R2>R3	R2>R3>R1	R2>R3>R1
C = C arom.	~1600	R3>R2>R1	R3>R2>R1	R3> R1> R2	R1>R2>R3	R1>R2>R3	R3>R2	R3>R2>R1	R3> R1> R2

^a^Absorbance intensities in R1, R2 and R3 ranges were read at temperatures designated on the basis of dDSC changes

In short, the presence of sharp bands between 4000–3500 cm^-1^ was assigned to differently bounded kinds of water [18,41] while low-intensity bands at 3500–3300 and 3020–3016 cm^-1^ were attributed respectively to the nitrogen compounds and aliphatic structures (including CH_4_) [42]. The main peak with the highest intensity, at 2400–2240 cm^-1^, originated from CO_2_ [18,41]. The bands around 1900–1650 cm^-1^ could be assigned to asymmetric stretching of C-O in carbonyl moieties from carboxylic groups, esters, ketones and aldehydes [9]. The absorption bands at around 1650–1530 cm^-1^ and 1550–1400 cm^-1^ were attributed to aromatic structures, however in the former range, carbonyl compounds could also have been detected [9], whereas in the latter, bending vibrations of aliphatic groups could have also overlapped. Confirmation of the carboxylic groups was found as symmetric stretching vibrations at the range of 1390–1250 cm^-1^. The alcohol groups of different order absorbed below 1200 cm^-1^.

**Fig 5:**
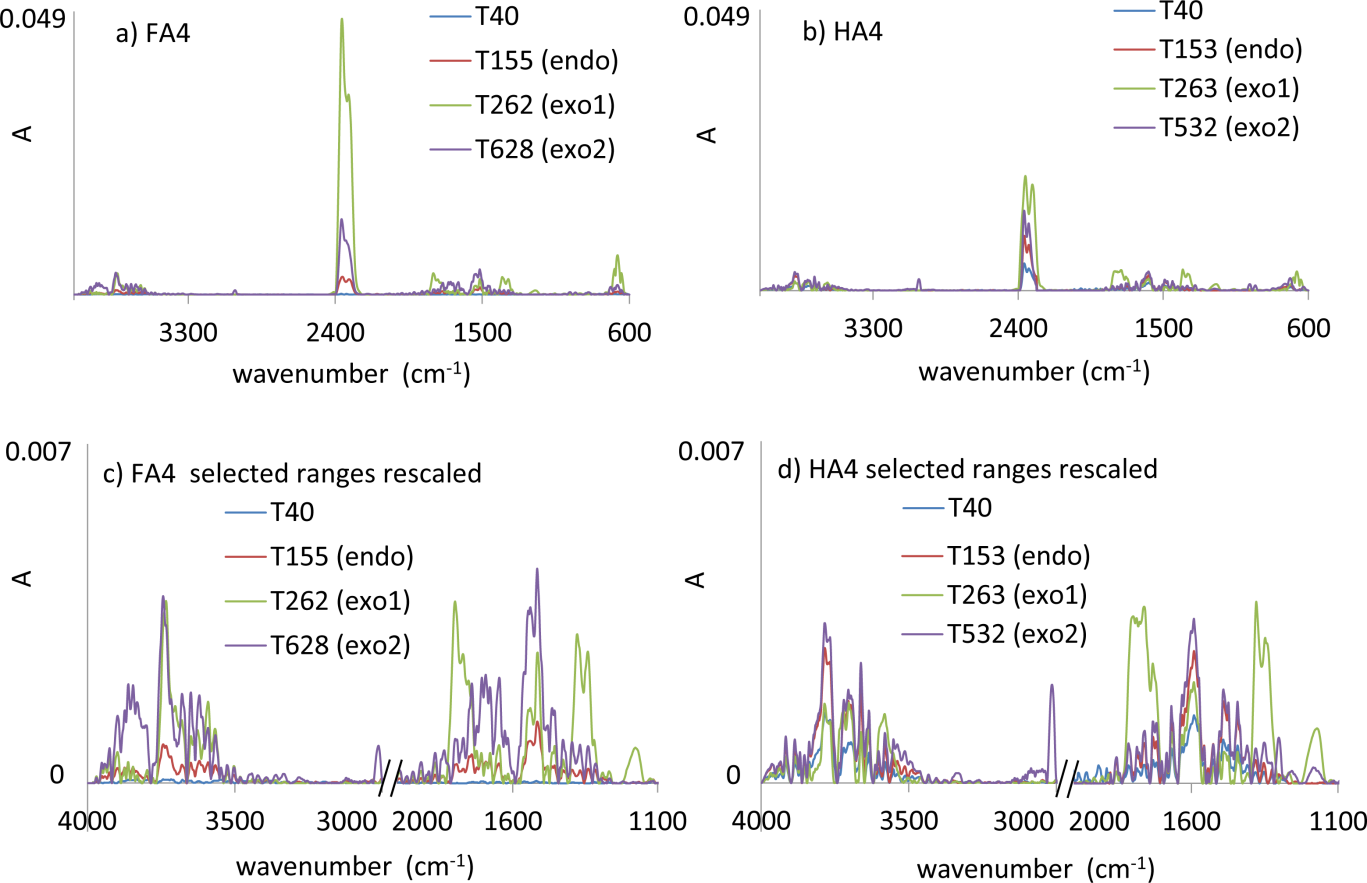
FTIR 2D spectra of FA4 (a, c) and HA4 (b, d) for characteristic temperatures of R1, R2 and R3 intervals.

In order to analyze the thermal decomposition process, 2D spectra were extracted from 3D spectra of each HA and FA sample from R1, R2 and R3 regions at temperatures of maximum of the DSC effect (determined according to dDSC changes). Exemplary 2D spectra for characteristic temperatures of R1, R2 and R3 intervals are overlapped and presented in Fig 5A–5D. The direction of absorption changes of main bands in R1, R2 and R3 temperature ranges are presented in Table 4. The results obtained from FTIR module provided additional data to the TG, DSC and QMS characteristics on gaseous products of pyrolytic decomposition. OH groups detected at ~3590 cm^-1^ showed the highest absorption at R2 region and the lowest at R1. This confirmed the presence of adsorbed water in R1 and decomposition of oxygen-containing functional groups in R2. The absorption intensities sequence R2>R3>R1 for this band was maintained for all HAs and FAs samples. OH absorption intensities at ~3780 cm^-1^ were in most cases the highest for the R3 region. Results obtained by Oudghiri et al. [33] on pyrolysis and combustion of marine sediment have confirmed the possibility of water releasing in such high temperatures. According to Liu et al. [20], absorption bands of this kind of water could be a result of intermolecular condensation reactions.

Aliphatic structures of the samples absorbed at about 3020–3016 cm^-1^ and seemed to be strongly thermally refractory due to their presence only at the R3 region (Fig 5C and 5D; Table 4). This assumption is in line with the statement of other researchers that the weight loss above 400°C for different organic materials is caused by slow and steady decomposition of the sp^2^-hybridized carbon skeleton [24,39,43] and with the findings of Arenillas et al. [19] that the formation of CH_4_ during coal pyrolysis reaches a maximum value at about 510°C. However, there are also some reports about decomposition of aliphatic groups at lower temperatures. In connection with the above, decomposition of aliphatic chains at such high temperatures seemed to be related to their length and chemical bonds with other structures [40]. Arenillas et al. [19] reported that release of CH_4_ may also originate from hydroaromatic and aryl methyl groups.

Emission of CO_2_ observed at ~2360 cm^-1^ showed the same temperature pattern for all studied samples and decreases in the temperature ranges in order: R2>R3>R1. These results corresponded with the area of m/z = 44 (CO_2_) obtained from the QMS module. Data from above two modules complemented one another. The area of m/z = 44 provided quantitative information on CO_2_ release, whereas FTIR 3D spectra of CO_2_ (Fig 3) revealed better-resolved peaks of thermal processes, indicating the presence of carbon compounds of different stability.

The order of absorbance of carbonyl compounds (1900–1650 cm^-1^) in temperature intervals was also the same as for CO_2_ (R2>R3>R1), however almost all decomposition of these structures took place at R2 temperatures. This indicated that only a small part of the carbonyls was connected with thermal refractory structures, degraded at R3 region. The C = O peaks were slightly higher for FAs than HAs, which indicated higher content of labile carboxylic groups in FAs. Exceptions were samples isolated from black earth, for which absorbance intensity of C = O was similar for both HA and FA. Aromatic compounds were mainly decomposed in the R3 region, which was exhibited in the FTIR 2D spectra (~1600 cm^-1^).

### Relationship between selected chemical properties of FAs and HAs and parameters of TG-DSC-QMS-FTIR analysis

**Fig 6:**
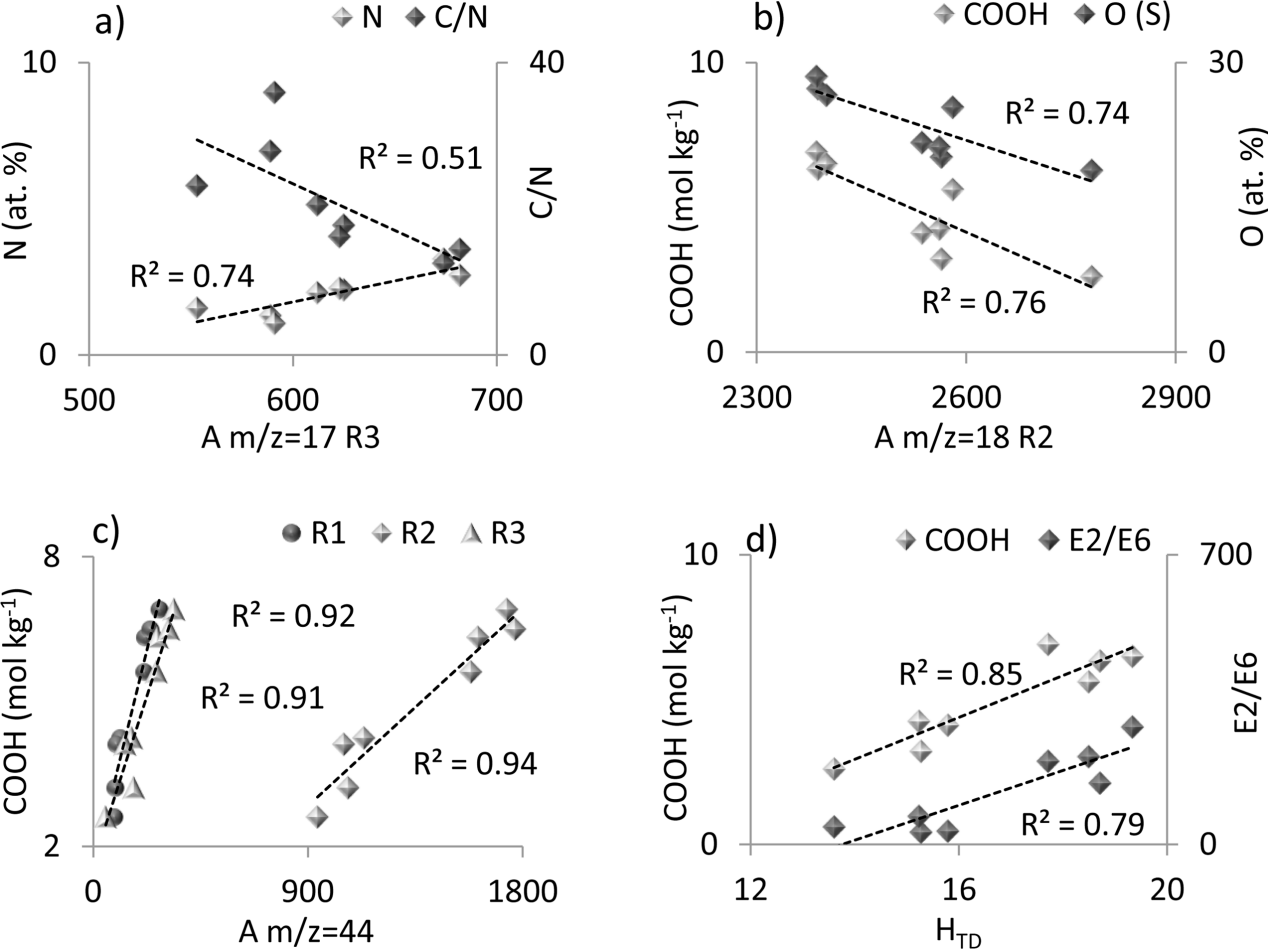
Exemplary relationships between TG-DSC-QMS-FTIR parameters and chemical properties of FAs and HAs (a) N content and C/N ratio vs. m/z = 17 area at R3; (b) COOH and O content vs. m/z = 18 area at R2; (c) COOH content vs. m/z = 44 area at R1, R2 and R3; (d) COOH content and E2/E6 ratio vs. H_TD_ index.

Statistical analysis of obtained results revealed relationships between some chemical properties of FAs and HAs and parameters calculated from TG-DSC-QMS-FTIR analysis. The matrix of correlation coefficients is presented in Table 5 and exemplary graphs in Fig 6A–6D.

**Table 5:** Coefficients of person’s correlations calculated between selected chemical properties of HAs and FAs and the parameters obtained from TG, DSC and MS. Bold digits highlight statistically significant relationships (p = 0.05).

	C	N	O (S)	C/N	COOH	OH	E2/E6	ΔlogK	E2/E4
**A m/z = 16 R1**	-0.54	-0.78	**0.97**	0.67	**0.95**	-0.69	**0.87**	**0.92**	**0.97**
**A m/z = 16 R2**	-0.53	**-0.78**	**0.96**	**0.76**	**0.96**	-0.51	**0.95**	**0.94**	**0.98**
**A m/z = 16 R3**	0.24	0.55	-0.60	-0.36	-0.51	0.73	-0.35	-0.41	-0.57
**A m/z = 17 R1**	-0.08	**-**0.71	0.70	0.66	0.67	**-0.72**	**0.79**	**0.79**	**0.86**
**A m/z = 17 R2**	**0.84**	0.65	**-0.91**	-0.60	**-0.93**	0.23	-0.71	-0.72	-0.77
**A m/z = 17 R3**	0.40	**0.86**	**-0.74**	**-0.71**	-0.69	0.53	-0.62	-0.58	-0.70
**A m/z = 18 R1**	-0.04	-0.64	0.58	0.53	0.52	-0.69	0.61	0.60	0.70
**A m/z = 18 R2**	**0.83**	0.57	**-0.86**	-0.56	**-0.87**	0.15	-0.62	-0.62	-0.69
**A m/z = 18 R3**	0.01	0.66	-0.57	-0.59	-0.47	0.50	-0.54	-0.51	-0.58
**A m/z = 44 R1**	-0.54	-0.76	**0.97**	0.68	**0.96**	-0.61	0.91	0.94	**0.98**
**A m/z = 44 R2**	-0.56	-0.76	**0.97**	0.72	**0.97**	-0.49	**0.95**	**0.95**	**0.97**
**A m/z = 44 R3**	-0.64	-0.61	**0.95**	0.59	**0.95**	-0.41	**0.88**	**0.88**	**0.92**
**Max**_**DSC**_ **R1**	-0.24	0.21	0.20	-0.12	0.18	0.42	0.22	0.20	0.05
**Max**_**DSC**_ **R2**	-0.01	**-0.77**	0.63	**0.83**	0.57	-0.40	0.67	0.64	0.66
**Max**_**DSC**_ **R3**	-0.15	-0.38	0.68	0.41	0.66	-0.40	**0.81**	**0.83**	**0.75**
**ΔM(R2)**	**-0.78**	-0.48	**0.82**	0.46	**0.86**	-0.11	**0.73**	0.74	0.70
**H**_**TD**_	-0.63	-0.70	**0.91**	0.68	**0.92**	-0.27	**0.89**	**0.88**	**0.84**

Obtained results showed that the parameters determined from coupled TG-DSC-QMS-FTIR analysis for the particular thermal steps (R1, R2, R3) remained in significant relation with sorption properties, reactivity and humification degree of HS fractions. Strong linear and positive relationships (R≥0.95) were found for the content of carboxylic structures (COOH) and oxygen (O) with areas of m/z = 16 and m/z = 44 at R1 and R2 temperature region. This fact exhibited that decomposition of carbon structures below 430°C were connected mainly with carboxylic group degradation. These conclusions were additionally confirmed by positive correlations of O and COOH content with weight loss, however only at R2. It was interesting that negative linear correlations of COOH and O contents were observed, with the area of m/z = 17 and m/z = 18, especially at R2 and R3 (example on the Fig 6B). These results suggested forming water molecules from other structures in polycondensation and dehydration reactions [20]. Moreover, in our opinion, spread of points around a straight line may raise some doubts about the importance of dependence despite the statistical significance of correlation coefficients; thus, we assume that this result may require additional research. The content of OH groups in the humic substances slightly corresponded with the parameters calculated from TG-DSC-QMS-FTIR analysis at a given temperature interval. This fact denoted considerable variation in the thermal stability of OH groups, resulting from the different structures in which OH groups were incorporated.

The E2/E6 and E2/E4 UV-Vis parameters of HAs and FAs were strongly positively correlated with the area of m/z = 16 (at R1, R2) and area of m/z = 44 (at R2, R3). The above results indicated that the increase in the amount of released gases: CO_2_, CH_4_, -NH_2_ below 430°C and CO_2_ at 430–650°C could be related to the decrease in humification degree, aromaticity and molecular weight of HS. The highest R coefficients were determined for E2/E4, which indicated a high dependency of the emitted gases amount with the ratio of lignin-like structures at initial humification stage to the structures moderately humified. Positive relationships were also found for E2/E6 and ΔlogK with Max_DSC_ at R2, which illustrated a decrease in HA and FA humification with the increase in the maximum of the heat at 220–430°C. Interestingly, the m/z = 17 area exhibited a positive relationship with N content and negative relationship with C/N ratio of HAs and FAs at the R3 temperature region (Fig 6A). These findings showed that nitrogen structures were thermally refractory [21]. The above conclusion was also confirmed by results of FTIR module.

The H_TD_ index proposed by us for thermal description of the humification stage proved to be a very good and easy way for expression of this process. It was revealed by high, positive correlation coefficient of H_TD_ with E2/E6 and E2/E4 parameters (R≥0.84) and with O and COOH content (R≥91) (Fig 6D). The H_TD_ index included temperature intervals R1, R2 and R3, which suggested that structures decomposing below 220°C could also affect humification. In conclusion, the H_TD_ parameter proposed in these studies seems to be promising in the context of the humification processes assessment.

## Conclusions

Studies on HS have been problematic due to high differentiation in HS structure, stability and solubility. Consequently, conventional methods of HS characterizing can be unsatisfactory and new analytical tools are still required. In this work, the unique combination of TG-DSC-QMS-FTIR techniques was used for the study of the chemical properties and thermal stability of soil HAs and FAs under controlled pyrolysis conditions. The obtained results revealed that TG-DSC-QMS-FTIR provides valuable quantitative and qualitative information on thermal behavior and the differences in the emission profiles of gaseous, volatile species of HS, depending on HS fraction and origin. DSC analysis has displayed a number of thermal processes related to presence of various chemical structures with diversified bond energies, and weight loss at temperature intervals of these processes was helpful information about percentage amount of individual structures in sample composition. Evolved gas analysis consisting of QMS and FTIR modules enabled chemical identification of labile, refractory and recalcitrant structures (by masses in QMS and by functional groups in FTIR) as well as semi-quantitative comparison of different pyrolysis species from various fractions of HS of different origin at temperature ranges determined from DSC analysis.

The statistical relationships found between thermal and chemical parameters indicated a high potential of TG-DSC-QMS-FTIR method for use in monitoring the changes of HS. Especially, content of COOH and O, as well as UV-VIS structural parameters E2/E6, ΔlogK and E2/E4 exhibited high correlations with the amount of released individual gaseous species and with weight loss of labile structures. The humification index H_TD_ proposed in this work revealed significant differentiation within the studied samples. Moreover, H_TD_ demonstrated positive relationships with E2/E6, E2/E4 parameters, O and COOH content, which can make it useful for description of HS structure.

To summarize, results of these studies show that coupled TG-DSC-QMS-FTIR technique can provide valuable data concerning comprehensive assessment of HS properties. This method, offering simultaneous and real-time measurement of thermal reactions and evolved gaseous species, could be an alternative to a number of different analytical procedures.

## Supporting information

S1 Fig Thermogravimetric curves (TG) with 1^st^ derivatives (dTG) of FA and HA nos. 1 and 3. (TIF)

S2 Fig Differential scanning calorimetry (DSC) with 1^st^ derivatives (dDSC) of FA nos. 1, 2 and 4. (TIF)

S3 Fig Differential scanning calorimetry (DSC) and 1^st^ derivatives (dDSC) of HA nos. 1, 2 and 4. (TIF)
