# Supplementary figures and images for: Use of thermal analysis coupled with differential scanning calorimetry, quadrupole mass spectrometry and infrared spectroscopy (TG-DSC-QMS-FTIR) to monitor chemical properties and thermal stability of fulvic and humic acids
Source: PLoS One. 2017 Dec 14;12(12):e0189653. doi: 10.1371/journal.pone.0189653 (PMC5730223; doi:10.1371/journal.pone.0189653)

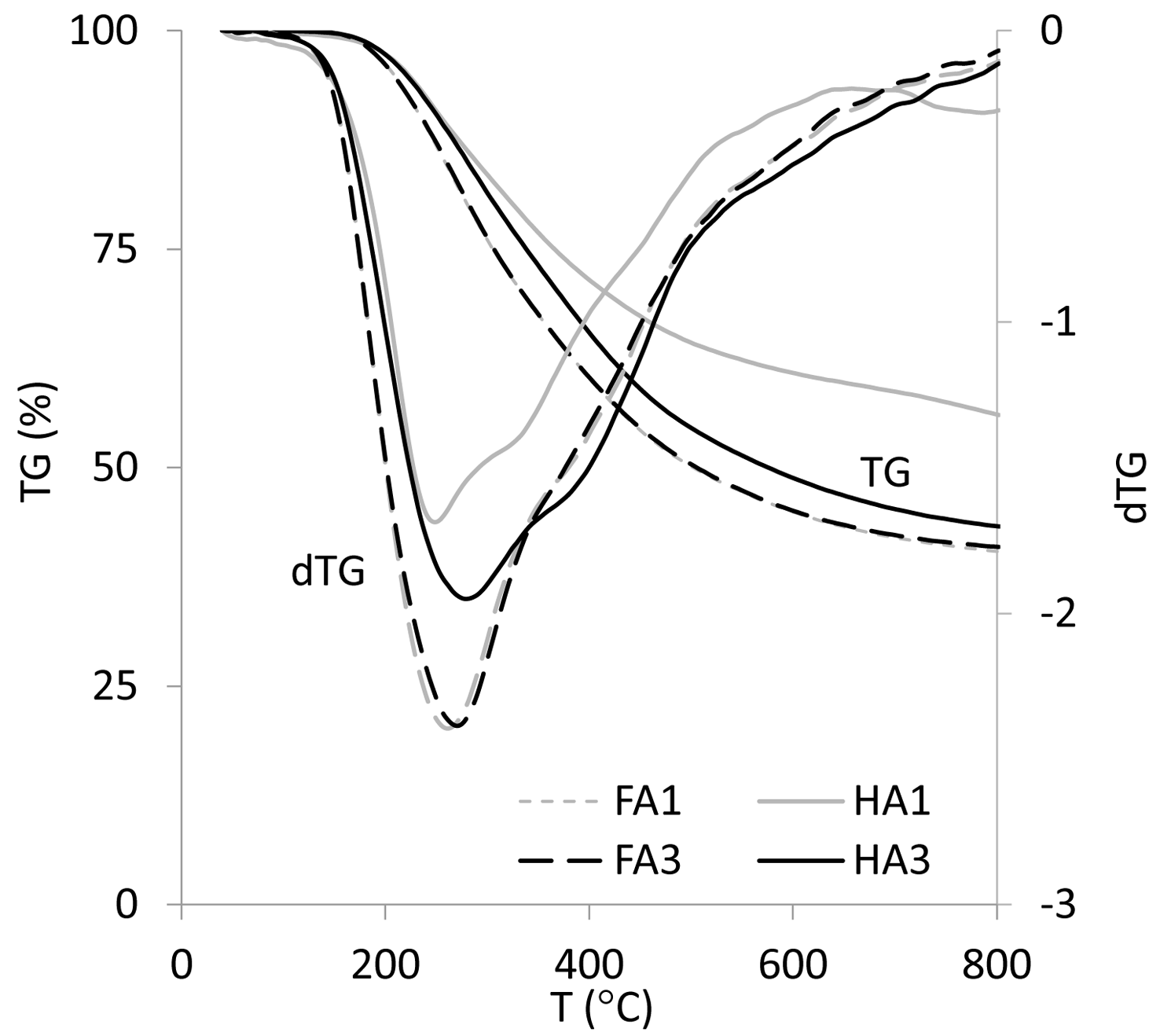

Supplement: S1 Fig — (TIF) [file pone.0189653.s001.tif]

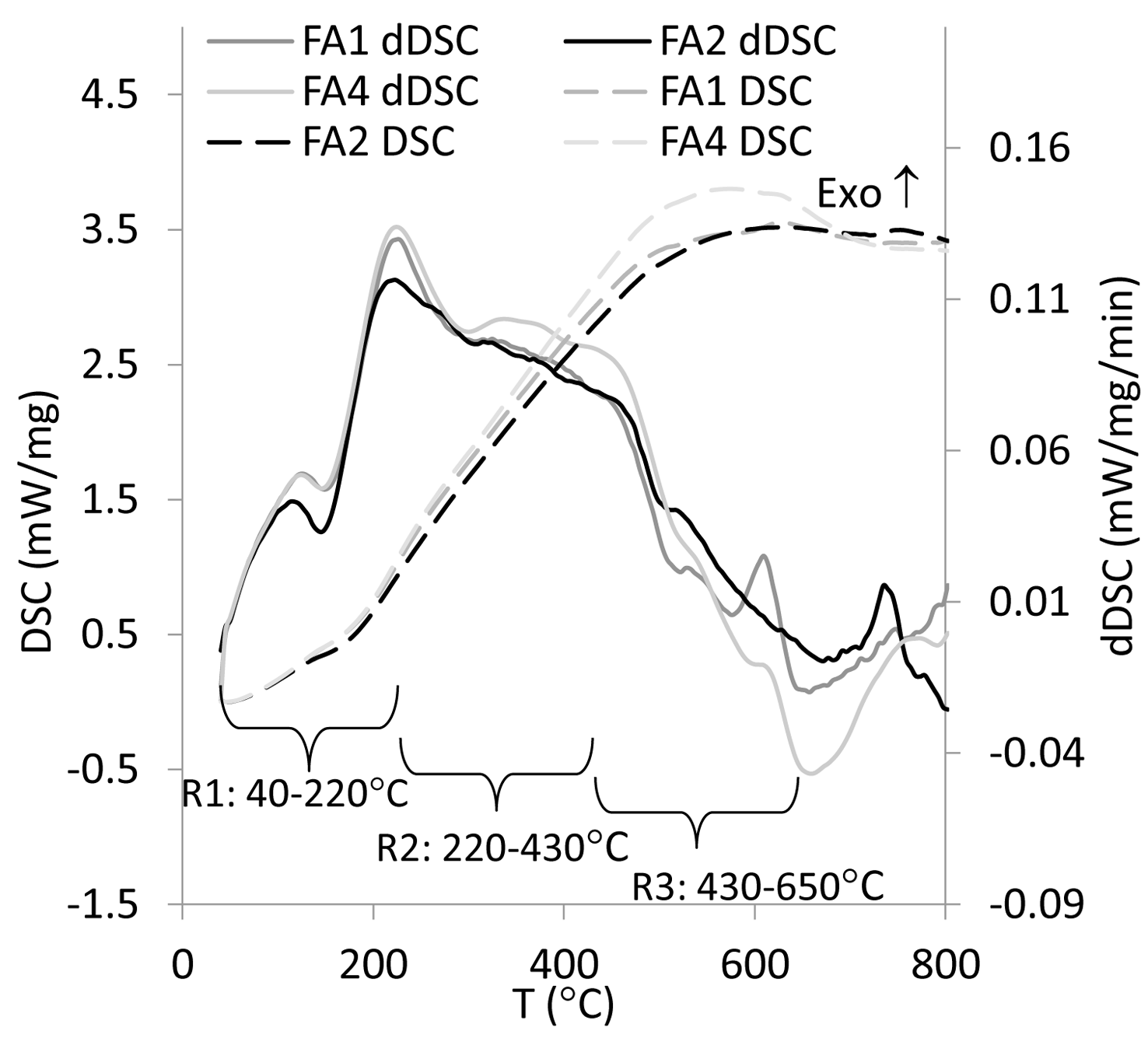

Supplement: S2 Fig — (TIF) [file pone.0189653.s002.tif]

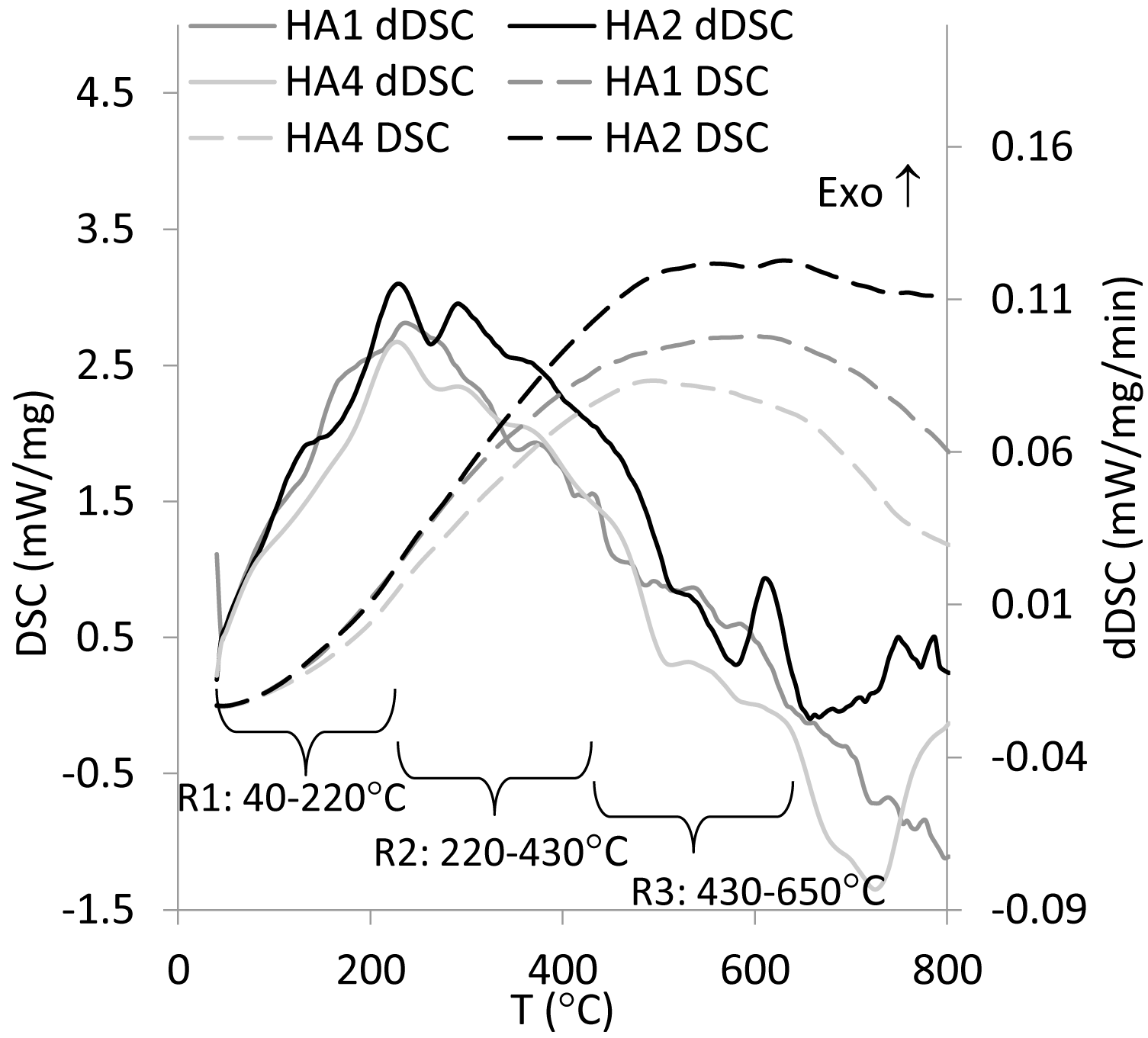

Supplement: S3 Fig — (TIF) [file pone.0189653.s003.tif]
